# Impact of heat generation/absorption of magnetohydrodynamics Oldroyd-B fluid impinging on an inclined stretching sheet with radiation

**DOI:** 10.1038/s41598-020-74787-2

**Published:** 2020-10-19

**Authors:** Fazle Mabood, Gabriella Bognár, Anum Shafiq

**Affiliations:** 1 Department of Information Technology, Fanshawe College London, London, ON Canada; 2 University of Miskolc, Miskolc-Egyetemváros, 3515 Hungary; 3 School of Mathematics and Statistics, Nanjing University of Information Science and Technology, Nanjing, 210044 China

**Keywords:** Engineering, Mechanical engineering

## Abstract

In this paper, we have investigated thermally stratified MHD flow of an Oldroyd-B fluid over an inclined stretching surface in the presence of heat generation/absorption. Similarity solutions for the transformed governing equations are obtained. The reduced equations are solved numerically using the Runge–Kutta Fehlberg method with shooting technique. The influences of various involved parameters on velocity profiles, temperature profiles, local skin friction, and local Nusselt number are discussed. Numerical values of local skin friction and local Nusselt number are computed. The significant outcomes of the study are that the velocity decreases when the radiation parameter $R_{d}$ is increased while the temperature profile is increased for higher values of radiation parameter $R_{d}$ in case of opposing flow, moreover, growth in Deborah number $\beta_{2}$ enhance the velocity and momentum boundary layer. The heat transfer rate is decrease due to magnetic strength but increase with the increased values of Prandtl and Deborah numbers. The results of this model are closely matched with the outputs available in the literature.

## Introduction

The non-Newtonian behaviour of liquids affects many chemical and manufacturing processes, particularly in material processing, nuclear and bioengineering. Due to numerous applications, the attention of a wide range of researchers have been attracted by the boundary layer flow of non-Newtonian fluids which are classified according to their behaviour in shear. The physical origin of non-Newtonian behaviour is related to the microstructure of the material. The viscosity of the generalized Newtonian fluid models depends on the shear rate (e.g. power-law, Carreau, Yasuda, Cross, Bingham, Herschel Bulkley, etc.). The Oldroyd-B model^1^ is a model with constant viscosity, which accounts both the relaxation and retardation times. Special examples of the Oldroyd fluid are the Maxwell fluid and viscous Newtonian fluid.

The Stokes’ first problem was extended for an Oldroyd-B fluid flow in a porous half space by Tan et al.^2^ using Fourier sine transform, and they gave an exact solution. Fetecau et al.^3^ discussed the energetic balance for the unsteady flow of an Oldroyd-B fluid driven by the transverse motion of an infinite plate subject to a time-dependent shear stress. In^4^, the hydromagnetic boundary layer flow of an Oldroyd-B fluid in a porous channel is investigated by using homotopy analysis method (HAM) when both suction and injection cases are considered.

During the past decades, there has been a growing recognition of the industrial significance of the magnetohydrodynamic (MHD) boundary layer flow of viscoelastic fluids over stretching surface, especially in petroleum and chemical engineering due to its applications in extraction of crude oil from petroleum products. Considerable efforts have been made to examine the MHD effects on different flow problems. For example, it is industrially important to discuss the motion of a fluid in a rotating or sliding cylinder in the oil exploitation. Jamil et al.^5^ investigated the velocity field and the shear stress corresponding to the motion of an Oldroyd-B fluid subject to torsional and longitudinal time-dependent shear stresses in a circular cylinder. Exact solutions are provided for the problem of the helical flows of a heated generalized Oldroyd-B fluid subject to a time-dependent shear stress in porous medium, where the motion is due to the longitudinal time-dependent shear stress and the oscillating velocity in boundary by Zheng et al.^6^.

Issues relating to the problem of fluid flow due to a stretching sheet have the applications in the field of the plastic film drawing. The first discussion on the fluid flow due to a stretched surface is given by Sakiadis^7^. A mathematical model for the two-dimensional Oldroyd–B fluid has been developed to describe the boundary layer in the region of the stagnation point over a stretching sheet by Sajid et al.^8^. The influence of rheological parameters in an Oldroyd-B fluid is discussed for three dimensional flows over a stretching surface by applying HAM in^9^, and over a stretching surface in the presence of convective boundary conditions in^10^.

The MHD flow over a nonlinear stretching sheet has been extensively studied because of its practical relevance in engineering applications. The variational iteration method has been used by Xu et al. to obtain approximate solutions of MHD boundary layer equations^11^. In magnetic field the viscoelastic properties of Oldroyd-B fluid through a planar channel were investigated on peristaltic flow by Hayat et al.^12^. Numerical simulations have been presented in the work^13^ to analyse the velocity, temperature and concentration profiles in MHD boundary layer flow over a linearly stretching sheet with viscous dissipation and second-order slip and to study the melting heat transfer of a water-based nanofluid. Mabood et al.^14^ conducted numerical results using similarity method to the governing equations of MHD flow when the surface is non-isothermal, stretched, suction or injection, and heat generation or absorption are considered. Several studies are devoted to the examination of the heat and mass transfer on MHD flow over a porous stretched surface using HAM, see e.g., Jitender et al.^15^.

Due to the heating and cooling industrial processes, the heat enhancement is important physical phenomena. The heat transfer properties of traditional coolants in heat exchangers can be increased with adding different nanoparticles to the base fluid. Ferrofluids having magnetic nanoparticles are useful in industrial applications. The effect of the ferromagnetic parameter on the flow and heat transport of the ferrofluids along a horizontal stretching sheet placed in a magnetic field is investigated in papers^16–18^. Similarity solution for boundary layer flow of non-Newtonian fluids over a stretching flat surface was reported by Bognar and Hriczo^19^. The stretching problem with power-law velocity was investigated in the flow of a non-Newtonian power-law fluid in the presence of uniform magnetic field in^20^. The impact of power-law surface velocity and temperature variation on the heat and mass transfer was given for two thermal boundary conditions of uniform surface heat flux and of varying surface temperature in^21^. The heat and mass transfer problems with chemical reactions are important in drying engineering processes. Analytic solutions using HAM are provided for the velocity, temperature and concentration distributions to study an MHD fluid flow over a stretching sheet when chemical reaction is in presence (see^22^).

Stratification of fluid arises due to temperature variations, concentration differences, or the presence of different fluids. It is an important issue to analyse the effect of thermal stratification on the flow properties as the heat and mass transfer mechanisms run parallel. Using HAM, the momentum and energy equations for thermally stratified flow of an Oldroyd-B fluid with mixed convection has been analysed in^23^, and for thermally stratified radiative flow of Maxwell fluid in^24^. Free convection heat and mass transfer problem in an electrically conducting micropolar fluid over a vertical plate with magnetic, thermal, and solutal stratification effects is solved in^25^. Waqas et al.^26^ have performed numerical results to the model of mixed convection for two-dimensional flow of Oldroyd-B liquid over a linearly stretching sheet. The solutions are obtained using homotopy method for the flow problem when thermal and concentration stratifications are considered. The solutions to mixed-convection flow of a thixotropic fluid over a linearly stretched surface are given by HAM in the presence of thermal stratified effects and thermal radiation by Shezad et al.^27^.

Based on the importance of nanomaterials, the flow characteristics are examined in the presence of internal heat generation^28^, with convection and radiation^29^ and in case of nonlinearly permeable stretching sheet with radiation^30^. The boundary layer flow over an inclined surface has been considered in papers^31–34^. Recently, the time dependent Darcy-Forchheimer fluid and the Oldroyd-B fluid were investigated due to slip condition^35^, over stretching sheet in the presence of uniform heat source or sink^36^ and with thermal radiation^37^.

In this paper, a model the thermal stratified MHD flow of Oldroyd-B fluid over an inclined linearly stretching sheet is considered. The skin friction and heat transfer characteristics are encountered. Furthermore, the thermal radiation effect is considered. Mathematical modelling is subjected to boundary layer assumptions and Roseland’s approximation. The governing nonlinear flow model is solved by Runge–Kutta Fehlberg method with shooting scheme. The impact of physical parameters of interest are elaborated. To our best knowledge this problem has not been reported before.

## Mathematical formulation

**Figure 1:**
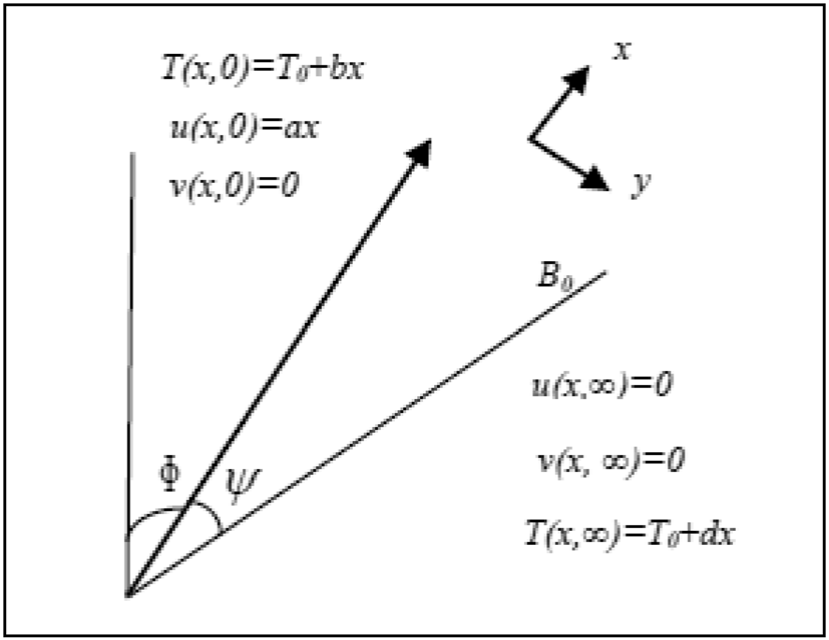
Schematic diagram.

We investigate the steady thermally stratified flow of an incompressible electrically conducting flow of an Oldroyd-B liquid along an inclined surface. Flow is generated because of stretched surface. The heat phenomenon is analysed by considering thermal radiation and heat absorption/generation. A uniform magnetic field *B*_*0*_ is taken inclined by making angle $\psi$ as shown in Fig. 1. The governing equations with above assumptions are^27^:

1 $$ \frac{\partial u}{{\partial x}} + \frac{\partial v}{{\partial y}} = 0 $$

2 $$ \begin{gathered} u\frac{\partial u}{{\partial x}} + v\frac{\partial u}{{\partial y}} + \lambda_{1} \left( {u^{2} \frac{{\partial^{2} u}}{{\partial x^{2} }} + 2uv\frac{{\partial^{2} u}}{\partial x\partial y} + v^{2} \frac{{\partial^{2} u}}{{\partial y^{2} }}} \right) = \nu \frac{{\partial^{2} u}}{{\partial y^{2} }} \hfill \\ + \nu \lambda_{2} \left( {u\frac{{\partial^{3} u}}{{\partial x\partial y^{2} }} + v\frac{{\partial^{3} u}}{{\partial y^{3} }} - \frac{\partial u}{{\partial y}}\frac{{\partial^{2} u}}{{\partial y^{2} }} - \frac{\partial u}{{\partial y}}\frac{{\partial^{2} v}}{{\partial y^{2} }}} \right) - \frac{{\sigma B_{0}^{2} }}{\rho }\sin^{2} \psi \left( {u + \lambda_{1} v\frac{\partial u}{{\partial y}}} \right) \hfill \\ + g\beta \sin \phi \left\{ {\left( {T - T_{\infty } } \right) + \lambda_{1} \left( {\frac{\partial T}{{\partial x}} + v\frac{\partial T}{{\partial y}} - \frac{\partial u}{{\partial x}}\left( {T - T_{\infty } } \right)} \right)} \right\}, \hfill \\ \end{gathered} $$

3 $$ u\frac{\partial T}{{\partial x}} + v\frac{\partial T}{{\partial y}} = \frac{1}{{\rho c_{p} }}\frac{{\partial^{2} T}}{{\partial y^{2} }} + \frac{Q}{{\rho c_{p} }}\left( {T - T_{\infty } } \right) - \frac{{\partial q_{r} }}{\partial y}\,, $$

subject to

$$ u = U_{w} \left( x \right) = ax,\,\,\,\,\,v = 0,\,\,\,\,T = T_{w} = T_{0} + bx\quad {\text{at}}\quad y = 0, $$

4 $$ u \to 0,\,\,\,\,\,T \to T_{\infty } = T_{0} + dx,\quad {\text{at}}\quad y \to \infty , $$

where the velocity components (*u, v*) in the *x* and *y*-directions are respectively, $\rho$ represents the density, *c*_*p*_ represents the specific heat, *k* represents the thermal conductivity, $a,\,\,b,$ and $d$ are constant such that $a > 0,\,\,b > 0,\,\,d = \frac{{dT_{\infty } }}{dx} > 0$*,* $\sigma$ represents the electrical conductivity, $\nu$ represents the kinematic viscosity, $Q$ represents heat source/sink, $\lambda_{1}$ ratio of the relaxation to retardation times, $g$ is the gravitational acceleration, $\lambda_{2}$ is the retardation time. Rosseland approximation of radiation of thick optical layer gives:

5 $$ q_{r} = - \frac{{4\sigma^{*} }}{{3k^{*} }}\frac{{\partial T^{4} }}{\partial y}, $$

where $\sigma^{*}$ and $k^{*}$ are Stefan-Boltzmann constant and mean absorption coefficient respectively and

$T^{4} \approx 4T_{\infty }^{3} T - 3T_{\infty }^{4}$ (linear combination of temperature).

Then, Eq. (3) takes the form:

6 $$ u\frac{\partial T}{{\partial x}} + v\frac{\partial T}{{\partial y}} = \frac{1}{{\rho c_{p} }}\left( {k + \frac{{16\sigma^{*} T_{\infty }^{3} }}{{3k^{*} }}} \right)\frac{{\partial^{2} T}}{{\partial y^{2} }} + \frac{Q}{{\rho c_{p} }}\left( {T - T_{\infty } } \right)\,. $$

We assume the following similarity transformation^7^:

7 $$ u = cxf^{\prime}\left( \eta \right),\,\,\,v = - \sqrt {c\nu } f\left( \eta \right),\,\,\,\eta = \sqrt {\frac{c}{\nu }} y,\,\,\,\,\theta = \frac{{T - T_{\infty } }}{{T_{w} - T_{0} }}\,, $$

Using Eq. (7), the nonlinear ordinary differential equations which represents the velocity and temperature profile can be reduced to

8 $$ \begin{gathered} f^{\prime\prime\prime} - f^{{\prime}{2}} + ff^{\prime\prime} + \beta_{1} \left( {2ff^{\prime}f^{\prime\prime} - f^{{\prime}{2}} f^{\prime\prime\prime}} \right) + \beta_{2} \left( {f^{{\prime\prime}{2}} - ff^{iv} } \right)\, - M^{2} \sin^{2} \psi \left( {f^{\prime} + \beta_{1} ff^{\prime\prime}} \right) \hfill \\ \,\,\,\,\,\,\,\,\,\,\,\,\,\,\,\,\,\,\,\,\,\,\,\,\,\,\,\,\,\,\,\,\,\,\,\,\,\,\,\,\,\,\,\,\,\,\,\,\,\,\,\,\,\,\,\,\,\,\,\,\,\,\,\,\,\,\,\,\,\,\,\,\,\,\,\,\,\,\,\,\,\,\,\,\,\,\,\,\,\,\,\,\,\,\,\,\,\,\,\,\,\,\,\,\,\,\,\,\,\,\,\,\,\,\,\,\,\,\,\,\,\,\,\,\,\,\,\,\,\,\, + \lambda \sin \phi \left( {\theta - \beta_{1} f\theta^{\prime}} \right) = 0\,, \hfill \\ \end{gathered} $$

9 $$ \left( {1 + \frac{4}{3}R_{d} } \right)\theta^{\prime\prime} - \Pr \left( {f\theta^{\prime} - f^{\prime}\theta + St\,f^{\prime}} \right) + \Pr \alpha \theta = 0\,, $$

where the boundary conditions take the dimensionless form:

10 $$ f\left( 0 \right) = 0\,,\,\,\,f^{\prime}\left( 0 \right) = 0,\,\,\,\theta \left( 0 \right) = 1 - St, $$

11 $$ f^{\prime}\left( \eta \right) \to 0\,,\,\,\,\theta \left( \eta \right) \to 0\,,\quad {\text{when}}\quad \eta \to \infty , $$

The dimensionless parameters are defined as:

$\beta_{1} = \lambda_{1} c$ (Deborah number in relaxation time, where c is constant), $\beta_{2} = \lambda_{2} c$ (Deborah number in retardation time), $\lambda = \frac{{Gr_{x} }}{{{\text{Re}}_{x}^{2} }}$ (mixed convection parameter), $Gr_{x} = \frac{{g\beta \left( {T - T_{0} } \right)x^{3} }}{{\nu^{2} }}$ (Grashof number^38^), ${\text{Re}}_{x} = \frac{{U_{w} \left( x \right)x}}{\nu }$ (local Reynolds number), $St = \frac{d}{b}$ (thermally stratified parameter), $\Pr = \frac{{\mu c_{p} }}{k}$ (Prandtl number), $\alpha = \frac{Q}{{\rho ac_{p} }}$ (heat source/sink parameter), $R_{d} = \frac{{4\sigma^{*} T_{\infty }^{3} }}{{3k_{\infty } }}$ (radiation parameter), $M^{2} = \frac{{\sigma B_{0}^{2} }}{\rho c}$ (magnetic parameter).

The skin friction coefficient *C*_*f*_ is defined as follows

$${C}_{f}=\frac{2{ \tau }_{w}}{\rho {U}_{w}^{2}},{ \tau }_{w}=\frac{2}{{U}_{w}^{2}}{c}^{3/2}f{^{\prime}}(0)$$

The local Nusselt number $Nu_{x}$ and the local heat flux ${q}_{w}$ are defined as:

12 $$ Nu_{x} = \frac{{xq_{w} }}{{k\left( {T_{w} - T_{0} } \right)}},\,\,\,\,q_{w} = - \left( {k + \frac{{16\sigma^{*} T_{\infty }^{3} }}{{3k^{*} }}} \right)\left( {\frac{\partial T}{{\partial y}}} \right)_{y = 0} . $$

The dimensionless form of local Nusselt number is:

13 $$ \frac{{Nu_{x} }}{{\sqrt {{\text{Re}}_{x} } }} = \frac{ - 1}{{\left( {1 - St} \right)}}\left( {1 + \frac{4}{3}R_{d} } \right)\theta^{\prime}\left( 0 \right). $$

## Method of solution

The closed form solutions of the reduced Eqs. (8) and (9) with boundary conditions (Eqs. 10, 11) are not possible to be achieved due to the fact that they are highly non-linear and coupled in nature. Nevertheless, their solutions could be achieved numerically using the Runge–Kutta–Fehlberg (RKF) with the shooting method that considers various values of parameters. Then, a study on the effects of the emerging parameters on the dimensionless velocity, temperature, and Nusselt number is carried out. The step size of $\Delta \eta = 0.01$, with the accuracy to be up to the fifth decimal place, is taken as the criterion of convergence.

## Results and discussion

In this section, the effect of various parameters on the velocity, temperature, and heat transfer rate is investigated. Tables 1, 2, 3 and 4 are provided for a relative study of current and previous limiting outcomes. Comparison of $f^{\prime\prime}\left( 0 \right)$ for various values of magnetic parameter $M$ when $\beta_{1} = \beta_{2} = \lambda = 0$ is shown in Table 1. It is seen that the obtained solution is in good agreement with those obtained by Xu and Le^1^, Mabood and Mastroberdino^2^, and Hayat et al.^3^. Table 2 is prepared to compare heat transfer rate with Mabood et al. ^4^ when $\beta_{2} = \lambda = \alpha = R_{d} = St = 0$ and $\Pr = 1$. Clearly, an acceptable agreement is noted. Table 3 shows the comparison of the heat transfer rate with Ali^11^ and Mabood et al.^12^ when $\beta_{1} = \beta_{2} = \lambda = \alpha = R_{d} = St = 0$ for various values of $\Pr$. It is noticed that there is a favourable matching between obtained and previous results in a limiting case. Table 4 represents the numerical values of $f^{\prime\prime}\left( 0 \right)$ and $- \theta^{\prime}\left( 0 \right)$ of the present analysis. It is observed that with the absence of magnetic field, the magnitude of $f^{\prime\prime}\left( 0 \right)$ increases with increasing Deborah number $\beta_{1}$ and $St$ while decreases for increasing $\beta_{2}$, $R_{d}$ and $\alpha$. Further, for $M = 1$ the magnitude of $f^{\prime\prime}\left( 0 \right)$ is enhanced for $\alpha$, $\beta_{1}$ and $St$. Influences of $\beta_{2}$ and $R_{d}$ on $\left|{f}^{^{\prime\prime} }\left(0\right)\right|$ is are opposite. On the other hand, the magnitude of $\theta^{\prime}\left( 0 \right)$ increase by increasing $\beta_{2}$ while it is decreasing with increasing $\alpha$,$\beta_{1}$, $R_{d}$ and $St$.

**Table 1:** Comparison of $f^{\prime\prime}\left( 0 \right)$ for various values of $M^{2}$ when $\alpha ={\beta }_{1}={\beta }_{2}=\lambda ={R}_{d}=St=0, \psi =\phi =\frac{\pi }{2}$ and $\Pr = 1$.

$M^{2}$	Xu &Lee ^1^	Mabood & Mastroberardino^2^	Hayat et al.^3^	Present
0		− 1.000008	− 1.00000	− 1.00000
1	− 1.41421	− 1.4142135	− 1.41421	− 1.41421
5	− 2.4494	− 2.4494897	− 2.44948	− 2.44949
10	− 3.3166	− 3.3166247	− 3.31662	− 3.31662
50	− 7.1414	− 7.1414284	− 7.14142	− 7.14143
100	− 10.0498	− 10.049875	− 10.04987	− 10.04987
500	− 22.38302	− 22.383029	− 22.38302	− 22.38303
1000		− 31.638584	− 31.63858	− 31.63858

**Table 2:** Comparison of $Nu_{x} \left( {{\text{Re}}_{x} } \right)^{ - 1/2}$ for various values of $M^{2}$ when $\Pr = 1$ $\alpha ={\beta }_{1}={\beta }_{2}=\lambda ={R}_{d}=St=0, \psi =\phi =\frac{\pi }{2}$.

$M^{2}$	Mabood et al.^4^	Present
5	0.67051	0.67051
10	0.54649	0.58201

**Table 3:** Comparison of heat transfer rate $- \theta^{\prime}\left( 0 \right)$ when $M=\alpha ={\beta }_{1}={\beta }_{2}=\lambda ={R}_{d}=St=0, \psi =\phi =\frac{\pi }{2}$.

$\Pr$	Ali^11^	Mabood et al.^12^	Present
0.72	0.8058	0.8088	0.80883
1	0.9691	1.0000	1.00000
3	1.9144	1.9237	1.92368
10	3.7006	3.7207	3.72067

**Table 4:** Numerical values of $f^{\prime\prime}\left( 0 \right)$ and $- \theta^{\prime}\left( 0 \right)$ with different parameters when $\psi = \phi = \frac{\pi }{2}$,$\mathit{Pr}=1, \lambda =0.2.$

$\downarrow$ Parameters $\to$					$M = 0$		$M = 1$	
$\alpha$	$\beta_{1}$	$\beta_{2}$	$R_{d}$	$St$	$f^{\prime\prime}\left( 0 \right)$	$- \theta^{\prime}\left( 0 \right)$	$f^{\prime\prime}\left( 0 \right)$	$- \theta^{\prime}\left( 0 \right)$
0.1	0.1	0.1	0.1	0.1	− 0.89143	0.86171	− 1.27305	0.75721
0.2					− 0.88720	0.80620	− 1.29311	0.87936
0.3					− 0.88131	0.74148	− 1.25668	0.53383
0.1	0.2				− 0.91194	0.85295	− 1.27699	0.75696
	0.3				− 0.93215	0.84455	− 1.28095	0.75672
	0.5				− 0.97168	0.82888	− 1.28891	0.75631
0.1	0.1	0.2			− 0.85654	0.87175	− 1.22148	0.77112
		0.3			− 0.82549	0.88085	− 1.17576	0.78371
		0.5			− 0.77250	0.89667	− 1.12682	1.09482
0.1	0.1	0.1	0.2		− 0.88694	0.80274	− 1.27009	0.69965
			0.4		− 0.87902	0.71019	− 1.26520	0.61025
			0.6		− 0.87227	0.64054	− 1.26135	0.54398
0.1	0.1	0.1	0.5	0.3	− 0.90611	0.61846	− 1.28691	0.52757
				0.6	− 0.95182	0.53163	− 1.32246	0.45432
				1	− 1.01197	0.40626	− 1.36963	0.35165

**Figure 2:**
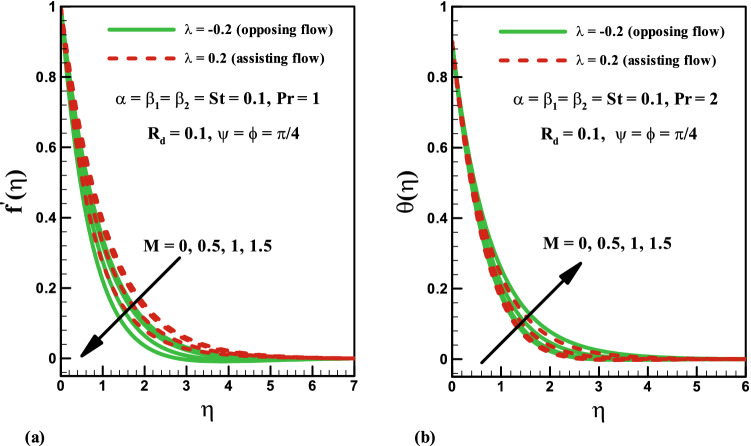
Effects of *M* on velocity and temperature for opposing and assisting flow.

**Figure 3:**
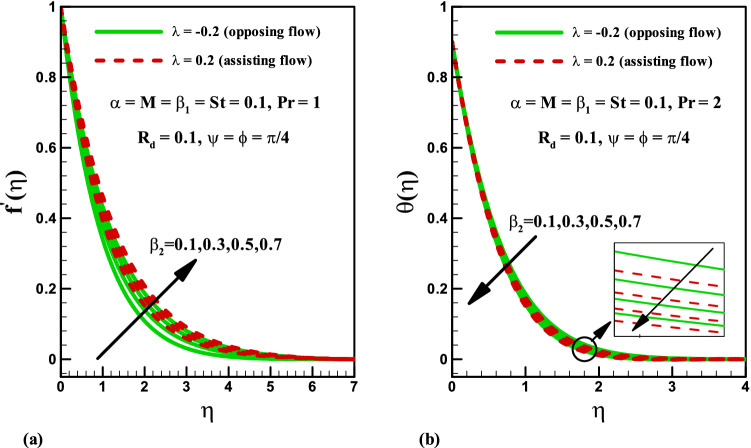
Effects of $\beta_{2}$ on velocity and temperature for opposing and assisting flow.

**Figure 4:**
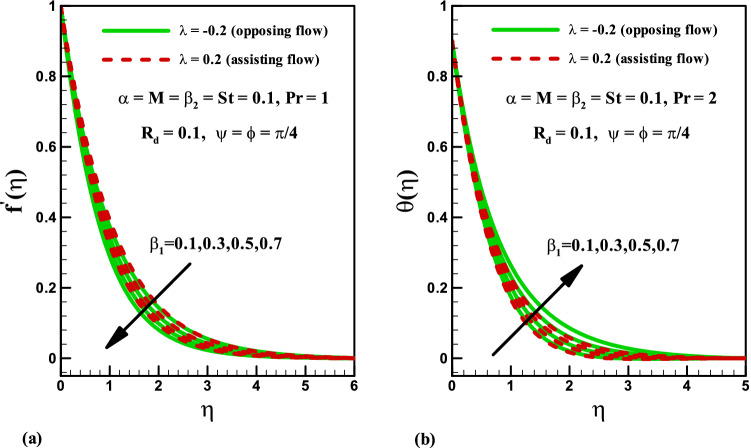
Effects of $\beta_{1}$ on velocity and temperature for opposing and assisting flow.

The significance of magnetic parameter $M$ on the velocity and temperature profiles for both opposing ($\lambda =-0.2$) and assisting ($\lambda =0.2$) flows is shown in Fig. 2a. It is noticed that velocity profile reduces via higher magnetic parameter $M$ for both cases (Fig. 2a). Physically, by increasing magnetic field the Lorentz force enhances. More resistance is observed to the motion of fluid and thus the velocity of liquid is reduced. However, opposite impact is observed for magnetic parameter $M$ on temperature distribution for both cases (see Fig. 2b). In fact, Lorentz force increases for higher magnetic number $M$ and consequently more heat is produced which give rise to temperature profile. Figure 3a is plotted for illustrating the impact of Deborah number $\beta_{2}$ (in terms of retardation time) on the velocity distribution for both opposing and assisting phenomena. It is concluded that for both cases the velocity and related boundary layer thickness are higher for increment in Deborah number $\beta_{2}$. The significance of Deborah number $\beta_{2}$ on temperature distribution is drawn in Fig. 3b. Temperature and thermal boundary thickness decrease by increasing $\beta_{2}$. Figure 4a is plotted for the variation of Deborah number in terms of relaxation time $\beta_{1}$ on velocity distribution in both opposing and assisting flow cases. It is observed that velocity distribution decreases with an increase in Deborah number $\beta_{1}$ for both the cases. Physically, the ratio of relaxation to observation time links to Deborah number ${\beta }_{1}$, so an enhancement in Deborah number means the greater relaxation time provides more resistance to the fluid motion which causes the reduction in velocity profile. Furthermore, the boundary layer thickness is higher for small values of $\beta_{1}$. The influence of Deborah number $\beta_{1}$ on temperature is displayed in Fig. 4b for both opposing and assisting flows. It is shown that temperature enhances with an increase in $\beta_{1}$ and thermal boundary layer also increases.

**Figure 5:**
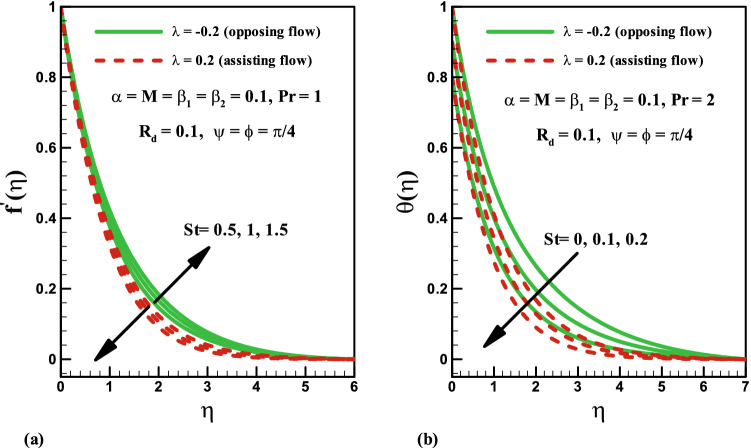
Effects of *St* on velocity and temperature for opposing and assisting flow.

Figure 5a shows the impact of thermal stratification parameter $St$ on the velocity distribution in both opposing and assisting flows. The result shows that the velocity and the associated boundary layer thickness decrease for larger values of thermal stratification parameter for assisting case, but the velocity increases for the opposing flow case. The density of the fluid in the lower region becomes greater than in the upper region for increasing values of $St$. Hence, thermal stratification $St$ slows down the convective flow between the heated surface and the ambient fluid, the velocity distribution decreases. The impact of the thermal stratification parameter $St$ on the temperature profile is presented in Fig. 5b for both cases. This indicates that the temperature and thermal boundary layer thickness decrease for both opposing and assisting cases with increasing values of $St$. It is because of the fact that the temperature difference gradually decreases between the surface of sheet and ambient fluid inducing a reduction in the temperature distribution.

**Figure 6:**
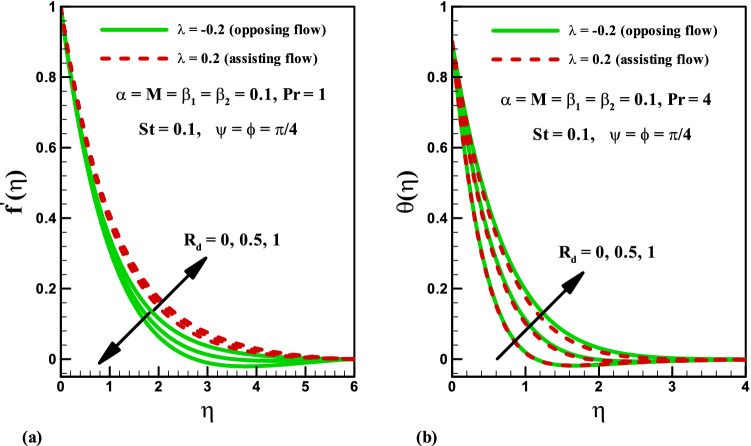
Effects of *R*_*d*_ on velocity and temperature for opposing and assisting flow.

Influence of radiation parameter $R_{d}$ on velocity distribution is plotted in Fig. 6a. Larger values of radiation parameter $R_{d}$ result in the enhancement of velocity field $f^{\prime}\left( \eta \right)$ for assisting case. Obviously, the velocity reduces in the opposing flow. Furthermore, the momentum boundary layer has opposite behaviour for both phenomena. Impact of radiation parameter $R_{d}$ on temperature distribution is presented in Fig. 6b for both opposing and assisting flows. Larger values of radiation parameter $R_{d}$ result in the enhancement of temperature field $\theta \left( \eta \right)$ for both cases. Physically, an increase in radiation parameter $R_{d}$ corresponds to lower mean absorption coefficient which is responsible for the enhancement of temperature distribution $\theta \left( \eta \right)$.

**Figure 7:**
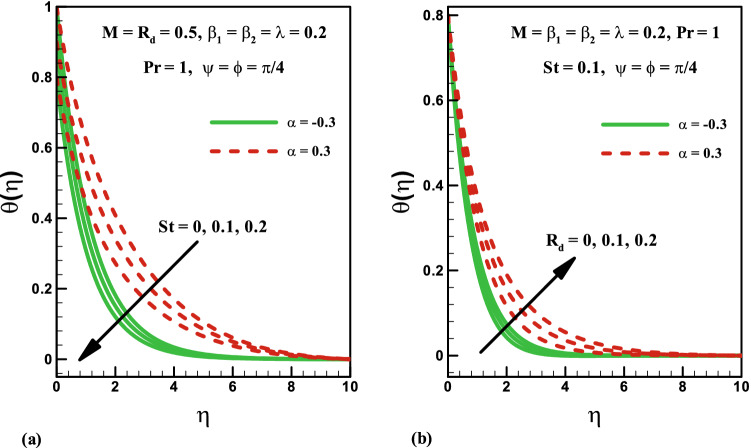
Effects of *St* and *R*_*d*_ on temperature for heat generation/absorption.

**Figure 8:**
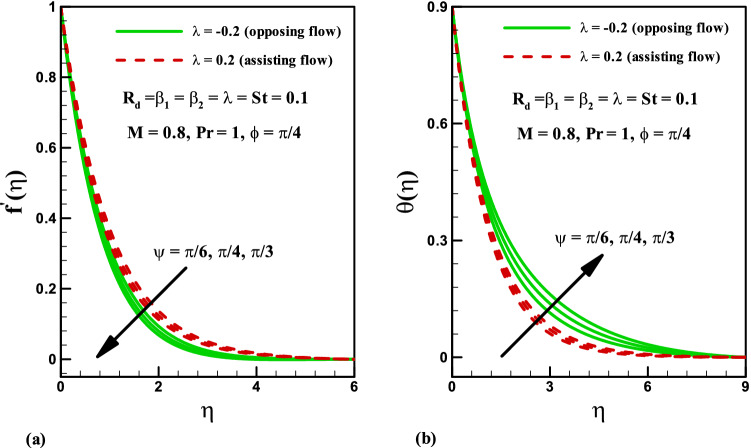
Effects of $\psi$ on velocity and temperature for opposing and assisting flow.

**Figure 9:**
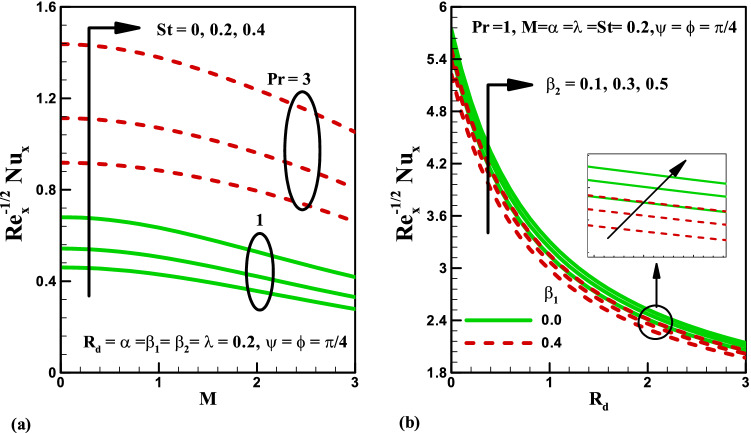
Effects of *St, Pr, M,* $\beta_{1}$*,* $\beta_{2}$ and* R*_*d*_ on local Nusselt number.

Variation of stratified parameter $St$ on temperature distribution is shown in Fig. 7a for the heat generation and absorption cases. Here both temperature and thermal boundary thickness decrease with the increase in stratified parameter $St$ = 0.0, 0.1, 0.2, 0.3 for the cases of heat generation and absorption. On the other hand, the variation of radiation parameter $R_{d}$ on temperature distribution is presented in Fig. 7b for the cases of heat generation and absorption. Temperature field is increased for higher values of radiation parameter $R_{d}$ for both heat generation/absorption cases. Actually, higher values of $R_{d}$ corresponds to lower mean absorption coefficient which is responsible for the enhancement of temperature distribution $\theta \left( \eta \right)$. Figure 8a,b illustrate the effect of inclination angle $\psi$ on velocity and temperature for both opposing and assisting flow respectively. It is noticed that the fluid velocity increase and temperature decrease with the increasing values of $\psi$. Figure 9 demonstrates the features of Prandtl number $\Pr$, thermal stratification parameter $St$, magnetic parameter $M$, radiation parameter $R_{d}$, and Deborah number $\beta_{2}$ on Nusselt number. It is analysed that the Nusselt number is higher for larger values of $\Pr ,\,\,St$ and $\beta_{2}$ while it decreases with the increase in $R_{d}$ and $M$.

**Figure 10:**
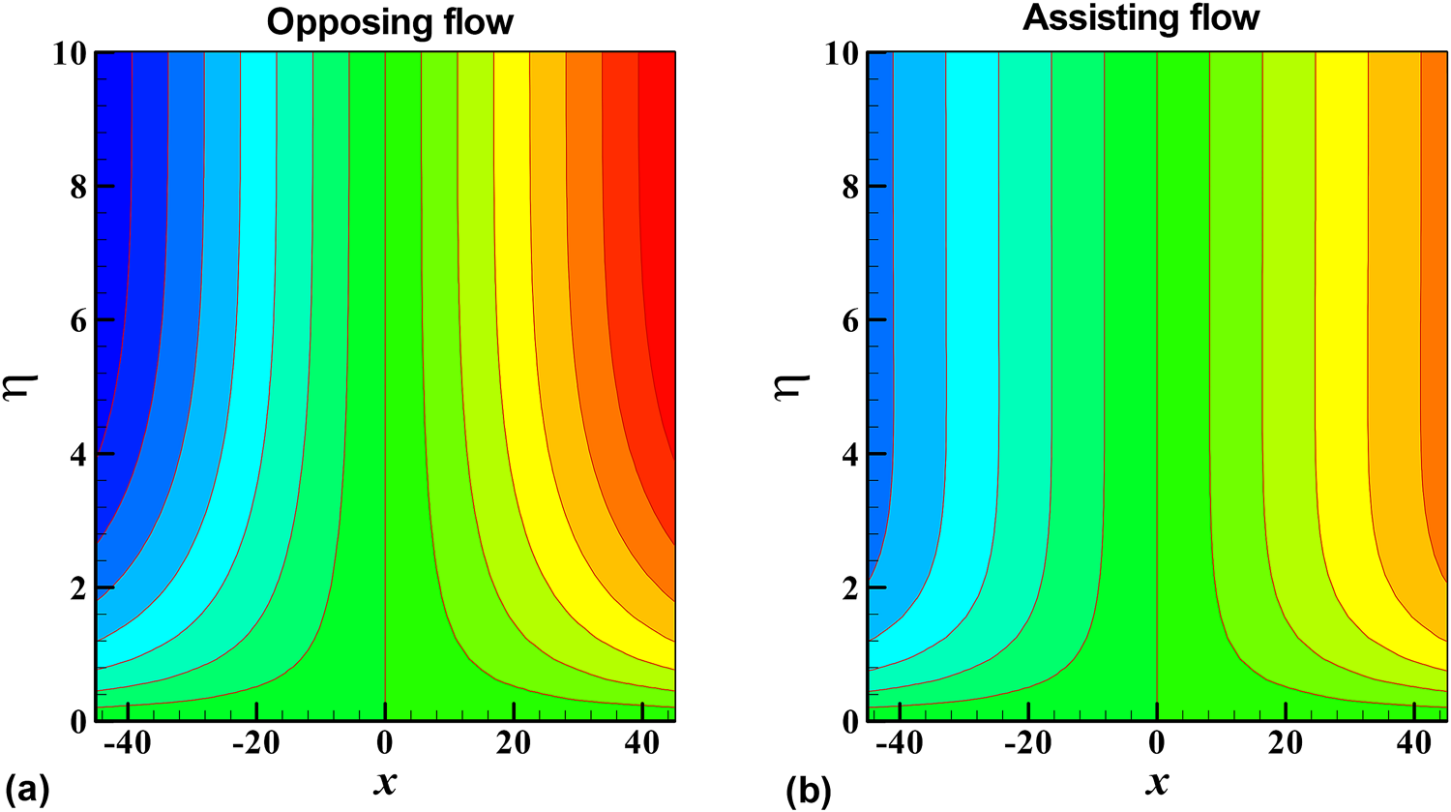
Contour plots of the streamlines for opposing and assisting flow.

**Figure 11:**
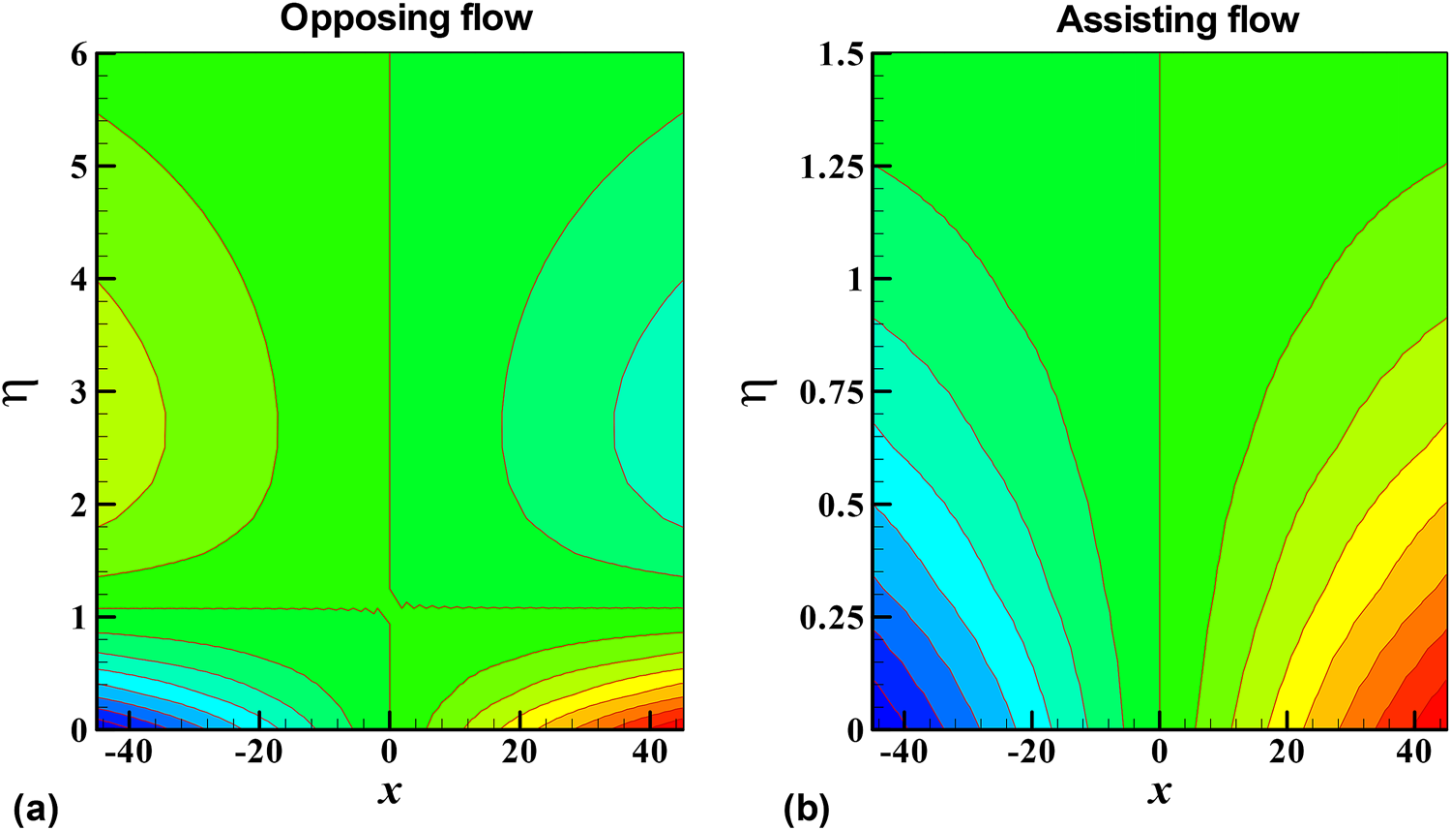
Contour plots of the isotherms for opposing and assisting flow.

Lastly, the streamlines and isotherms pattern are on display in Figs. 10 and 11, it is observed that streamlines/isotherms diverge/converge more and more from/to an origin in opposing flow as compared to assisting flow accordingly.

## Conclusions

The effect of heat source/sink in the thermally stratified MHD flow of an Oldroyd-B fluid over an inclined stretching surface is discussed. The outcomes of the present investigations are as follows.

- Velocity profile decreases with an increase in $St$ for opposing flow while opposite behaviour is observed for the assisting phenomenon.
- Deborah number $\beta_{1}$ increases the velocity profile for both opposing and assisting flows. However, the temperature decreases by increasing Deborah number $\beta_{1}$ for both cases.
- Velocity is an increasing function of impact of Deborah number $\beta_{2}$.
- Temperature and associated boundary layer thickness are increasing functions of radiation parameter.
- Velocity profile decreases when radiation parameter $R_{d}$ is increased while temperature profile is increased for higher values of radiation parameter $R_{d}$ in case of opposing flow.
- The Nusselt number is higher for greater values of $\Pr ,\,\,St,\,\,R_{d}$ and $\beta_{2}$ while it decreases with an increase in $M$.
- In the real life application, the Oldroyd-B fluid model corresponds to study the behavior of the flow of blood through an abdominal aortic segment (hemodynamics).

## List of symbols

$$T_{\infty }$$$$\left( {\text{K}} \right)$$: Ambient fluid temperature
$$g$$$$\left( {{\text{m}}\,{\text{s}}^{ - 2} } \right)$$: Acceleration due to gravity
$$a,\,\,b,\,\,c,\,\,d$$: Constants
$$\rho$$$$\left( {{\text{kg}}\,{\text{m}}^{ - 3} } \right)$$: Density of the fluid
$$\mu$$$$\left( {kg\,m^{ - 1} s^{ - 1} } \right)$$: Dynamic viscosity of the fluid
$$\sigma$$$$\left( {S\,m^{ - 1} } \right)$$: Electrical conductivity
$$T$$$$\left( K \right)$$: Fluid temperature in the boundary layer
$\alpha$: Heat source/sink
$$\psi$$: Inclination/angle
$$\nu$$$$\left( {{\text{m}}^{2} {\text{s}}^{ - 1} } \right)$$: Kinematic viscosity of the fluid
$$q_{w}$$$$\left( {{\text{Cal}}{.}\,{\text{m}}^{ - 2} {\text{s}}^{ - 1} } \right)$$: Local heat flux
${\text{Re}}_{x}$: Local Reynolds number
$Nu_{x} {\text{Re}}_{x}^{{ - \,\,\frac{1}{2}}}$: Local Nusselt number
$$M^{2}$$: Magnetic parameter
$k^{*}$: Mean absorption coefficient
$\lambda$: Mixed convection parameter
$$\Pr$$: Prandtl number
$$R_{d}$$: Radiation parameter
$$c_{p}$$$$\left( {{\text{J}}\,{\text{kg}}^{ - 1} {\text{K}}^{ - 1} } \right)$$: Specific heat
$\sigma^{*}$: Stefan–Boltzmann constant
$Gr$: Thermal Grashof number
$$k$$$$\left( {{\text{W}}\,\,{\text{m}}^{ - 1} \,{\text{K}}^{ - 1} } \right)$$: Thermal conductivity
$St$: Thermally stratified parameter
$$T_{w}$$$$\left( {\text{K}} \right)$$: Temperature on the surface
$B_{0}$: Uniform strength of magnetic field
$$(u,v)$$$$\left( {{\text{m}}\,{\text{s}}^{ - 1} } \right)$$: Velocity components along (x, y) directions

## Subscripts

$w$: Quantities at wall
$\infty$: Quantities at free stream

## References

[1] Oldroyd JG (1950). On the formulation of rheological equations of state. Proc. R. Soc. Lond..

[2] Tan W, Takashi M (2005). Stokes first problem for an Oldroyd-B fluid in a porous half space. Phys. Fluids.

[3] Fetecau C, Zierep J, Bohning R, Fetecau C (2010). On the energetic balance for the flow of an Oldroyd-B fluid due to a flat plate subject to a time-dependent shear stress. Comput. Math. Appl..

[4] Hayat T, Shehzad SA, Mustafa M, Hendi A (2012). MHD flow of an Oldroyd-B fluid through a porous channel. Int. J. Chem. Reactor Eng..

[5] Jamil M, Fetecau C, Imran M (2011). Unsteady helical flows of Oldroyd-B fluids. Commun. Nonlinear Sci. Numer. Simulat..

[6] Li C, Zheng L, Zhang Y, Ma L, Zhang X (2012). Helical flows of a heated generalized Oldroyd-B fluid subject to a time dependent shear stress in porous media. Commun. Nonlinear Sci. Numer. Simul..

[7] Sakiadis, B. C. Boundary layer behaviour on continuous moving solid surfaces. I. Boundary layer equations for two-dimensional and axisymmetric flow. II. Boundary layer on a continuous flat surface. III. Boundary layer on a continuous cylindrical surface. *AIChE J*. **7** 26–28 (1961).

[8] Sajid M, Abbas Z, Javed T, Ali N (2010). Boundary layer flow of an Oldroyd-B fluid in the region of stagnation point over a stretching sheet. Can. J. Phys..

[9] Hayat T, Awais M, Obaidat S (2012). Similar solutions for three-dimensional flow in an Oldroyd-B fluid over a stretching surface. Numer. Methods Fluids.

[10] Hayat T, Shehzad SA, Alsaedi A, Alhothuali MS (2013). Three-dimensional flow of Oldroyd-B fluid over surface with convective boundary conditions. Appl. Math. Mech.-Engl. Ed..

[11] Xu L, Lee EWM (2013). Variational iteration method for the magnetohydrodynamic flow over a nonlinear stretching sheet. Abstr. Appl. Anal..

[12] Hayat T, Noreen S, Obaidat S (2012). Peristaltic motion of an Oldroyd-B fluid with induced magnetic field. Chem. Eng. Commun..

[13] Mabood F, Mastroberardino A (2015). Melting heat transfer on MHD convective flow of a nanofluid over a stretching sheet with viscous dissipation and second order slip. J. Taiwan Inst. Chem. Eng..

[14] Mabood F, Khan WA, Ismail AIM (2015). Approximate analytical modelling of heat and mass transfer in hydromagnetic flow over a non-isothermal stretched surface with heat generation/absorption and transpiration. J. Taiwan Inst. Chem. Eng..

[15] Jitender S, Mahabaleshwar US, Bognár G (2019). Mass transpiration in nonlinear MHD flow due to porous stretching sheet. Sci. Rep..

[16] Bognár G, Hriczó K (2019). Ferrofluid flow in the presence of magnetic dipole. Tech. Mech..

[17] Bognár, G., Hriczó, K., Szávai, Sz. & Stojanovic, B. Ferrofluid flow in the presence of magnetic field above stretching sheet. *Tribol. Indus.* **41** 426–432 (2019). 10.24874/ti.2019.41.03.12

[18] Bognár G, Hriczó K (2018). Similarity transformation approach for a heated ferrofluid flow in the presence of magnetic field. EJQTDE.

[19] Bognár G, Hriczó K (2016). On similarity solutions of MHD flow over a nonlinear stretching surface in non-Newtonian power-law fluid. EJQTDE.

[20] Bognár, G. Magnetohydrodynamic flow of a power-law fluid over a stretching sheet with a power-law velocity. in *Springer Proceedings in Mathematics and Statistics* 130–139, 10.1007/978-3-319-32857-7_13 (2016).

[21] Ali ME (1994). Heat transfer characteristics of a continuous stretching surface. Wärme-und Stoffübertragung.

[22] Mabood F, Khan WA, Ismail AIM (2015). MHD stagnation point flow and heat transfer impinging on stretching sheet with chemical reaction and transpiration. Chem. Eng. J..

[23] Hayat T, Hussain Z, Farooq M, Alsaedi A, Obaid M (2014). Thermally stratified stagnation point flow of an Oldroyd-B Fluid. Int. J. Nonlinear Sci. Numer. Simul..

[24] Hayat T, Shehzad SA, Al-Sulami HHS (2013). Asghar, Influence of thermal stratification on the radiative flow of Maxwell fluid. J. Braz. Soc. Mech. Sci. Eng..

[25] Srinivasacharya D, Upendar M (2013). Effect of double stratification on MHD free convection in a micropolar fluid. J. Egypt Math. Soc..

[26] Waqas M, Khan MI, Hayat T, Alsaedi A (2017). Stratified flow of an Oldroyd-B nanoliquid with heat generation. Results Phys..

[27] Shehzad SA, Qasim M, Alsaedi A, Hayat T, Alhuthali MS (2013). Combined effects of thermal stratification and thermal radiation in mixed convection flow of thixotropic fluid. Eur. Phys. J. Plus..

[28] Sowmya G, Gireesha BJ, Sindhu S, Prasannakumara BC (2020). Investigation of Ti6Al4V and AA7075 alloy embedded nanofluid flow over longitudinal porous fin in the presence of internal heat generation and convective condition. Commun. Theor. Phys..

[29] Baslem A (2020). Analysis of thermal behavior of a porous fin fully wetted with nanofluids: Convection and radiation. J. Mol. Liq..

[30] Gireesha BJ, Umeshaiah M, Prasannakumara BC, Shashikumar NS, Archana M (2020). Impact of nonlinear thermal radiation on magnetohydrodynamic three dimensional boundary layer flow of Jeffrey nanofluid over a nonlinearly permeable stretching sheet. Phys. A.

[31] Anwar, M. I., Rafque, K., Misiran, M., Shehzad, S. A.·& Ramesh, G.K. Keller box analysis of inclination flow of magnetized Williamson nanofluid. SN Appl. Sci. **2**, 377 (2020). 10.1007/s42452-020-2029-4

[32] Ramesh, G.K., Chamka, A. J. & Gireesha. Boundary layer flow past an inclined stationary/moving flat plate with convective boundary condition. *Afr. Mat.* **27**, 87–95 (2016). 10.1007/s13370-015-0323-x

[33] Ramesh GK, Chamka AJ, Gireesha BJ (2013). MHD mixed convection viscoelastic fluid over an inclined surface with a non-uniform heat source/sink. Can. J. Phys..

[34] Ramesh, G.K., Gireesha, B.J. & Bagewadi, C. S. Heat transfer in MHD dusty boundary layer flow over an inclined stretching sheet with non-uniform heat source/sink.* Adv. Math. Phys.* **657805**, 1–13 (2012). 10.1155/2012/657805

[35] Do Y, Ramesh GK, Roopa GS, Sankar M (2019). Navier’s slip condition on time dependent Darcy-Forchheimer nanofluid using spectral relaxation method. J. Cent. South Univ..

[36] Gireesha BJ, Ganesh Kumar K, Ramesh GK, Prasannakumara BC (2018). Nonlinear convective heat and mass transfer of Oldroyd-B nanofluid over a stretching sheet in the presence of uniform heat source/sink. Results Phys..

[37] Ganesh Kumar K, Ramesh GK, Gireesha BJ, Gorla RSR (2017). Characteristics of Joule heating and viscous dissipation on three-dimensional flow of Oldroyd B nanofluid with thermal radiation. Alex. Eng. J..

[38] Hayat T, Qayyum S, Alsaedi A, Asghar S (2017). Radiation effects on the mixed convection flow induced by an inclined stretching cylinder with non-uniform heat source/sink. PLoS ONE.

